# LIM domain-containing proteins Zyxin and LPP localize to apical epithelial cell-cell junctions at regions of stable F-actin

**DOI:** 10.17912/micropub.biology.002233

**Published:** 2026-08-31

**Authors:** Katherine K. Tjoelker, Yashar Bashirzadeh, Leah M. Beel, Allen P. Liu, Ann L. Miller

**Affiliations:** 1 Cellular & Molecular Biology Graduate Program, University of Michigan, Ann Arbor, MI; 2 Sharpixel LLC, Johnston, IA; 3 Department of Molecular, Cellular, and Developmental Biology, University of Michigan, Ann Arbor, MI; 4 Department of Mechanical Engineering, University of Michigan, Ann Arbor, MI

## Abstract

LIM domain-containing proteins, Zyxin and Lipoma-Preferred Partner (LPP), localize to sites where actin filaments are strained, including actin stress fibers, focal adhesions, and cell-cell junctions. Utilizing the *Xenopus laevis* embryonic epithelium and live confocal microscopy, this study demonstrates that Zyxin and LPP localize to apical cell-cell junctions in polarized epithelia. Furthermore, particle image velocimetry (PIV) analysis reveals an inverse correlation between Zyxin/LPP intensity and F-actin velocity, suggesting that Zyxin and LPP are present at regions of stable F-actin.

**Figure 1. Fluorescently-tagged Zyxin and LPP localize to apical cell-cell junctions at regions of stable F-actin:** (A) Zyxin and LPP have similar domain diagrams with an α-actinin binding domain, ActA repeats that bind to VASP, and three tandem LIM domains forming the LIM domain-containing region (LCR). (B) *Xenopus laevis* Zyxin and LPP’s major domains have 20.6-50.8% sequence identity with the LIM domains being the most conserved region. (C) Zyxin and LPP (Fire lookup table (LUT)), colocalize with ZO-1 (grayscale), and F-actin (Green Fire Blue LUT) at apical cell-cell junctions in *Xenopus laevis* embryos. Scale bars: 20 µm. (D, F) Side view and line scan analysis showing normalized intensity of ZO-1 (blue), PLEKHA7 (gold), Zyxin (purple), and F-actin (green) from the apical to basolateral side. Scale bar: 2 µm. Shaded area shows standard error of the mean. Intensity was normalized by dividing the intensity values by the average intensity of the lowest 20% of the values per channel. n = 140 junctions, 20 videos, 3 clutches of embryos. (E, G) Side view and line scan analysis showing normalized intensity of ZO-1 (blue), LPP (lavender), and F-actin (green) from the apical to basolateral side. Scale bar: 2 µm. Shaded area shows standard error of the mean. Intensity was normalized by dividing the intensity values by the average intensity of the lowest 20% of the values per channel. n = 105 junctions, 15 videos, 3 clutches of embryos. (H-J) Representative images of embryo where Zyxin-mNeonGreen and F-actin probe (Lifeact-miRFP703) were expressed. (H) Particle tracking of representative F-actin pixels overlayed on F-actin image. (I) F-actin intensity (LUT) and F-actin velocity (arrows show the velocity and direction of movement) for the representative image. (J) Zyxin intensity (LUT) and F-actin velocity (arrows) for the representative image. Enlargements highlight an area with high Zyxin intensity and low F-actin velocity (top) and an area with low Zyxin intensity and high F-actin velocity (bottom). (K) Zyxin intensity (left) and F-actin velocity (right) heat maps of the representative image (same as in H-J). Zyxin intensity heatmap (4×4-pixel square blocks, top left) and downsampled Zyxin intensity heatmap of the same image (16×16-pixel square blocks, bottom left). F-actin velocity heatmap (4×4-pixel square blocks, top right) and downsampled F-actin velocity heatmap of the same image (16×16-pixel square blocks, bottom right). (L) LPP intensity (left) and F-actin velocity (right) heat maps of a representative image. LPP intensity heatmap (4×4-pixel square blocks, top left) and downsampled LPP intensity heatmap of the same representative image (16×16-pixel square blocks, bottom left). F-actin velocity heatmap (4×4-pixel square blocks, top right) and downsampled F-actin velocity heatmap of the same representative image (16×16-pixel square blocks, bottom right).

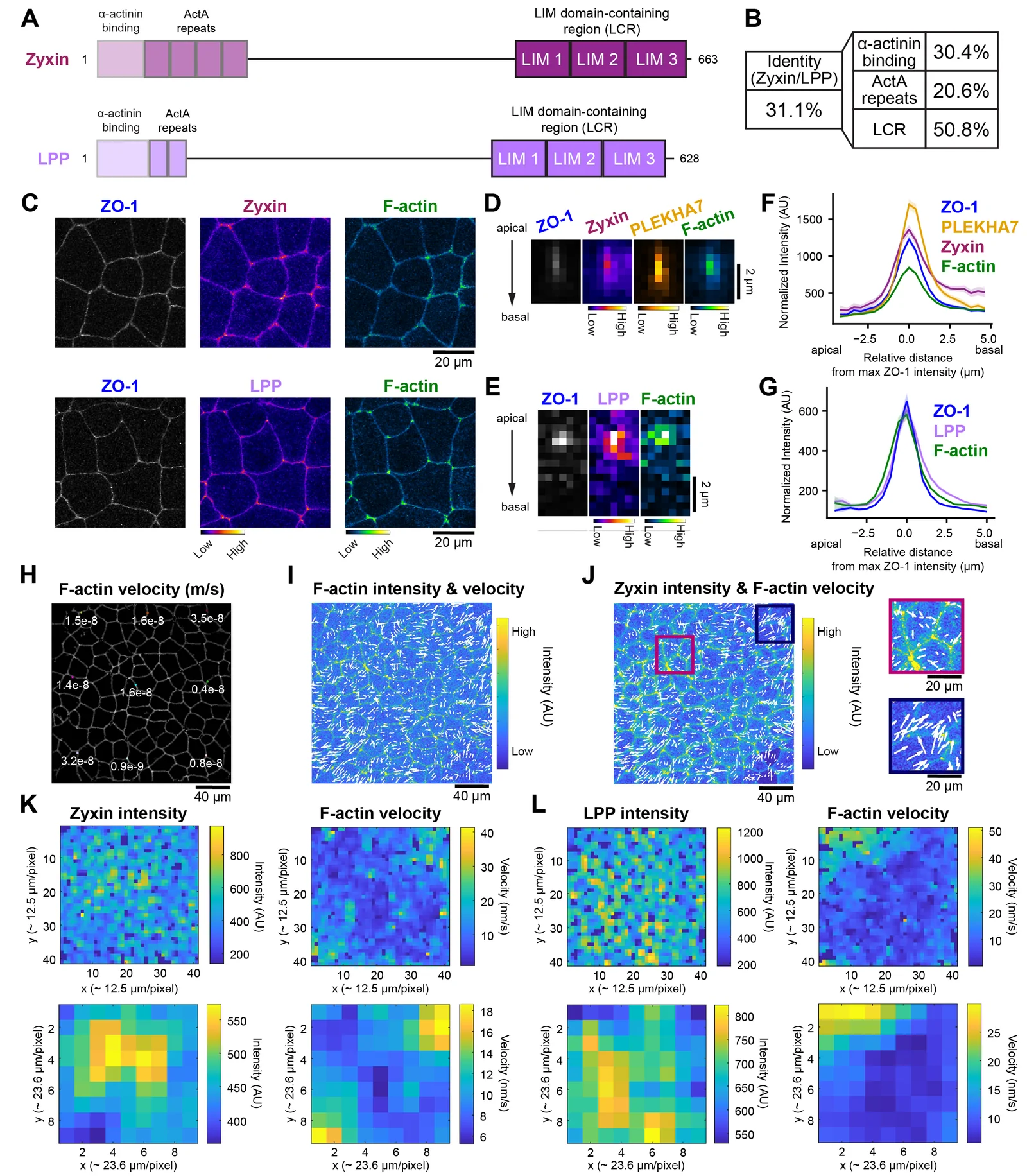



## Description

The actomyosin cytoskeleton plays important regulatory roles at cell-cell junctions and cell-matrix adhesions by generating forces and responding to forces acting on cells. These functions are key for cellular behaviors such as cell migration, cell-cell signaling, and embryo development (Kraning-Rush et al. 2011; Lakk et al. 2021; Kim et al. 2014). Actin-binding proteins support actomyosin’s roles in these processes by mediating actin polymerization, crosslinking, remodeling, and transmission of force to transmembrane proteins.

LIM domain-containing proteins are a mechanosensitive family of proteins that localize to actin stress fibers, focal adhesions, and cell-cell junctions (Anderson et al. 2021; Siddiqui et al. 2021). Within the Zyxin family of LIM domain-containing proteins, Zyxin and Lipoma-Preferred Partner (LPP) share similar domain maps ( **Figure 1A** ); however, their major domains share only 20-50% sequence identity ( **Figure 1B** ). The function and mechanism of each of Zyxin’s domains have been well characterized in the context of actin stress fibers, which connect to focal adhesions (Oakes, 2025). In contrast, less is known about the contribution of LPP’s domains to the protein’s function (Hansen & Beckerle 2006). In the context of focal adhesions, Zyxin accumulates at damaged actin stress fibers through its three LIM domains (Sun et al. 2020; Winkelman et al. 2020), which sense strain sites in actin filaments (Zsolnay et al. 2024). The LIM domains reinforce and stabilize F-actin through mechanosensitive recruitment to strained F-actin enabled by conserved phenylalanines in each LIM domain (Sun et al. 2020). Zyxin then recruits α-actinin (bundles actin) and VASP (helps actin polymerize) to repair damaged actin filaments (Hoffman et al. 2012).

Zyxin and LPP have also been studied in epithelial and epithelial-like cells. Epithelial cells are connected by cell-cell junctions, forming cohesive sheets that separate specialized compartments in the body. Adherens junctions (AJs) adhere epithelial cells to each other, while tight junctions (TJs) generate barrier function to selectively regulate tissue permeability. The actomyosin cytoskeleton, comprised of actin filaments (F-actin) and Myosin II, interacts with proteins at both TJs and AJs to transmit forces to and provide structural support for junctions (Arnold et al. 2017). In primary mouse keratinocytes that are forming new cell-cell junctions, Zyxin and VASP colocalize with E-cadherin at the tips of actin-rich filopodia as new AJs are forming (Vasioukhin et al., 2000). In MDCK cells in the early stages of forming cell-cell contacts, Zyxin and LPP both localize to the ends of actin bundles that terminate at cell-cell junctions (Hansen & Beckerle 2006). Notably, these types of actin bundles often occur both at newly forming cell-cell junctions and at vertices where multiple cells come together. Zyxin has also been studied in the developing epithelium of gastrula-stage *C. elegans* embryos and in the follicular epithelium of *Drosophila* ovaries (Lynch et al. 2022; Slabodnick et al. 2023; Jacobs et al. 2025). In *C. elegans* embryos, Zyxin localizes at apical junctions, which simultaneously serve the role of both TJs and AJs (Lynch et al. 2022). In the *Drosophila* epithelium, Zyxin localizes strongly at tricellular vertices, the junctions where three cells meet (Jacobs et al. 2025). Of note, *Drosophila* epithelial cells contain AJs and septate junctions, which perform a similar functional role to TJs, but are composed of different proteins.

A proximity biotinylation experiment carried out in MDCK II cells identified LPP as proximal to ZO-1, a cytoplasmic TJ protein that links transmembrane TJ proteins to F-actin (Van Itallie et al. 2013). Although MDCK II cells are commonly used as a model of polarized epithelia when cultured on permeable transwell filters, when MDCK II cells are grown on rigid surfaces, such as glass and plastic, the cells remain quite flat and fail to fully polarize (Hagelaars et al. 2022). In the proximity biotinylation experiment showing that LPP and ZO-1 are in close proximity, the MDCK II cells were cultured on plastic cell culture dishes and thus were not fully polarized (Van Itallie et al. 2013).

Altogether, these studies suggest that Zyxin localizes to AJs as new junctions form, and that Zyxin and LPP localize to apical cell-cell junctions in epithelia. Whether these LIM domain-containing proteins also associate with apical cell-cell junctions in vertebrate epithelial tissue remains unknown. Here, we examine fluorescently-tagged *Xenopus laevis* Zyxin and LPP localization in the *Xenopus* embryonic epithelium to determine their localization with respect to a TJ protein, an AJ protein, and to F-actin dynamics in polarized vertebrate epithelial cells.

We first live-imaged *Xenopus laevis* Zyxin-mNeonGreen or LPP-mNeonGreen along with a TJ marker (mRFP-ZO-1) and an F-actin probe (Lifeact-miRFP703) in polarized epithelial cells in the animal cap of gastrula-stage *Xenopus laevis* embryos ( **Figure 1C** ). We observe that Zyxin and LPP localize to cell-cell junctions and are strongly accumulated at cell vertices ( **Figure 1C** ). Side views from confocal z-stacks show that Zyxin and LPP are enriched at the apical surface in a similar pattern to ZO-1 and F-actin ( **Figure 1D- E** ). Line scan quantification of Zyxin-mNeonGreen or LPP-mNeonGreen intensity averaged over multiple junctions shows that Zyxin/LPP fluorescent intensity overlaps with ZO-1 and F-actin at the apical surface of epithelial cells ( **Figure 1F- G** ). Zyxin was also co-imaged with ZO-1 (TagBFP-ZO-1) and PLEKHA7 (PLEKHA7-mCherry), an AJ marker that localizes strongly at the zonula adherens (Shah et al. 2016). Although the signals of the TJ and AJ markers overlap, the Zyxin intensity peak closely overlapped with the ZO-1 peak, while it was slightly offset from the PLEKHA7 peak ( **Figure 1F** ). This data provides evidence that Zyxin and LPP localize to the F-actin associated with apical cell-cell junctions in the *Xenopus laevis* embryonic epithelium.

We then used particle image velocimetry (PIV) to measure changes in Zyxin/LPP intensity and F-actin velocity ( **Figure 1H- L** ) from live-imaging movies. Our data demonstrate an inverse relationship between Zyxin or LPP recruitment and F-actin velocity: high Zyxin/LPP intensity correlates with low F-actin velocity. Low F-actin velocity suggests that the F-actin is stable, so we tracked F-actin velocity ( **Figure 1H** ). We did not observe a trend between F-actin intensity and F-actin velocity ( **Figure 1I** ). We then plotted Zyxin intensity with an overlay of arrows showing the direction and speed of F-actin movement ( **Figure 1J** ). There is a noticeable relationship between high Zyxin intensity and low F-actin velocity. This trend becomes more striking when LIM protein intensity and F-actin velocity heatmaps are viewed side by side. We downsampled heatmaps to most clearly visualize the trend ( **Figure 1K- L** ).

To quantify the inverse relationship between Zyxin/LPP intensity and F-actin velocity across 10 examples for Zyxin and 15 examples for LPP, we identified the region with the highest 20% of Zyxin/LPP intensity and calculated the probability that the lowest 20% of F-actin velocity also occurred in that region. We then did the same for the lowest 20% of Zyxin/LPP intensity with the highest 20% of F-actin velocity. Using these cutoffs, any probability greater than 0.2 is indicative of a correlation between Zyxin/LPP intensity and F-actin velocity. We found that in regions where Zyxin or LPP intensity is high, there is a greater than random probability that F-actin velocity was low (P(V _low_ |Zyxin_I _high_ ) = 0.33±0.04; P(V _low_ |LPP_I _high_ ) = 0.39±0.02). The opposite was true for LPP but not Zyxin: in regions where LPP intensity was low, there was a higher than random probability that F-actin velocity was high (P(V _high_ |Zyxin_I _high low_ ) = 0.23±0.02; P(V _high_ |LPP_I _high low_ ) = 0.31±0.03). Since low F-actin velocity suggests that the F-actin is stable, these results indicate that Zyxin and LPP are found at stable F-actin. Future work is needed to determine whether Zyxin and LPP stabilize F-actin in these regions or are recruited to regions where stable F-actin is present.

In summary, we demonstrate that Zyxin and LPP localize to apical cell-cell junctions at the sites of stable F-actin in the developing epithelium of *Xenopus laevis* embryos. Further work will be needed to identify whether this is a result of Zyxin and LPP stabilizing F-actin. The regulation of junction-associated F-actin helps maintain junction integrity and barrier function. LPP knockdown was shown to slow the formation of barrier function in polarized MDCK II cells (Van Itallie et al. 2014). Since F-actin may be strained during the formation of new junctions, this result suggests that Zyxin and LPP may help stabilize F-actin and support processes that involve heightened mechanical forces, like the formation of new cell-cell junctions or the maintenance and remodeling of tricellular vertices.

## Methods

Plasmids and mRNA Preparation:

pCS2+/Zyxin-mNeonGreen and pCS2+/LPP-mNeonGreen were synthesized by Twist Bioscience after designing the plasmids using sequences from NM_001098681.1 (Zyxin.S) and NM_001096546.1 (LPP.L). mRNA was transcribed *in vitro* by first linearizing pCS2+-based DNA constructs using Not1-HF. pCSf107mT/Lifeact-miRFP703 was not linearized. The DNA constructs were then transcribed with the mMessage mMachine SP6 Transcription Kit (Invitrogen) and purified with the RNeasy Mini Kit (Qiagen). mRNA was stored at -80ºC until use.

*In vitro* fertilization and microinjections:

*In vitro* fertilization was performed using eggs collected from adult female frogs that were hyperovulated with human chorionic gonadotropin (MP Biomedicals) and testes harvested from male frogs. After dejellying fertilized embryos using 2% cysteine, pH 7.8 in 1X Mark’s Modified Ringer’s solution (MMR), 5 nl mRNA was injected into the animal hemisphere of the embryos four times at either the 2-cell or 4-cell stage. Each 5 nl injection contained the following amount of mRNA: 10 pg pCS2+/Zyxin-mNeonGreen or 10 pg pCS2+/LPP-mNeonGreen; 150 pg pCSf107mT/Lifeact-miRFP703; 125 pg pCS2+/mRFP-ZO-1; 70 pg pCS2+/TagBFP-ZO-1; 28 pg pCS2+/PLEKHA7-mCherry. Embryos in 0.1X MMR were mounted in a 0.8 mm-thick a metal slide with a ~5 mm hole in the center by holding them in place with two coverslips attached to the slide with vacuum grease.

All animal procedures strictly adhere to the compliance standards of the US Department of Health and Human Services Guide for the Care and Use of Laboratory Animals and were approved by the Institutional Animal Care and Use Committees at the University of Michigan. A board-certified laboratory animal veterinarian oversees our animal facility.

Live Imaging:

Videos were captured using an inverted Olympus FluoView 1000 confocal microscope with mFV-10-ASW software. Videos for Figure 1D- F were captured using an inverted Olympus FluoView 3000 confocal microscope with FV31S-SW software. For both microscopes, a supercorrected Plan Apo N 60XOSC objective (NA = 1.4, working distance = 0.12 mm) was used. Embryos were mounted in a chamber in a metal slide and held in place between two coverslips attached with vacuum grease.

Apical to Basolateral Intensity Analysis:

The Zyxin dataset includes 20 videos (1 video per embryo) from 3 clutches of embryos. The LPP dataset includes 15 videos (1 video per embryo) from 3 clutches of embryos. For seven bicellular junctions (BCJs) for a single time point in each video, a 5 µm line was drawn perpendicular to the BCJ in FIJI. The orthogonal view of this line was generated, and a second 1-pixel wide line was drawn from the apical side of the image to the basal side at the center of the junction. The intensity along this line was calculated for each channel. The intensity for each channel was normalized by dividing the intensity values by the average intensity of the lowest 20% of the values per channel. In order to overlay the line scans, the distance was normalized by offsetting the distance values so that 0 µm for each image was the location in the line scan where ZO-1 intensity was at its maximum. The mean intensity and standard error of the mean were plotted as a function of relative distance.

PIV and Correlation Analysis:

The LPP dataset described above and a Zyxin dataset that includes 10 videos (1 video per embryo) from 3 clutches of embryos were used. Cell segmentation was performed using a custom-developed image processing routine in MATLAB (MathWorks, Natick, MA). Cellular boundaries were detected from Zyxin and LPP intensity images to analyze their respective pixel intensities at individual cell-cell junctions within the dynamic tissue at each time point.

Particle image velocimetry (PIV) was employed to capture the displacement and velocity vector field of cell boundaries from actin images. To achieve this, a MATLAB algorithm was developed for batch processing of tissue dynamics using PIVlab source code material (Thielicke 2022; Thielicke et al. 2021). PIV algorithm was Multi-pass Fast Fourier Transform (FFT) window deformation with 4 passes at interrogation window sizes of 64, 32, 16, and 8 in order. Each pass had an interrogation window overlap of 50%. Post-processing vector validation was performed with standard deviation filter threshold of 8 and local median filter threshold of 3.

To generate Figure 1H, the Manual Tracking plugin of Fiji was used to measure displacement and speed of the labeled points on the representative image pair.

To generate Figures 1I-J, the velocity vector field of F-actin at the cell-cell junctions was mapped onto the intensity heatmap of F-actin (1I) and Zyxin (1J). Half of the measured velocity vectors are displayed in the figure panels for better visualization.

Velocity field resolution is determined by the final pass of PIV interrogation which generated an 85×85 velocity matrix. Zyxin or LPP intensity distribution within each interrogation window was averaged to scale down intensity image to 85 × 85 pix ^2^ thus assigning a protein intensity value to each velocity vector. This enabled us to derive an inverse relationship between Zyxin or LPP recruitment and F-actin dynamics ( **Figure 1K- L** ).

To obtain the spatial extent of statistical correlation between protein intensity and peripheral velocity, we first developed an algorithm that divided the velocity matrix into square blocks of 4×4 and 16×16 with 50% block overlap and derived mean velocity magnitude and mean Zyxin or LPP intensity heatmaps within the blocks ( **Figure 1K- L** ). We then measured the probability of high velocity magnitude (80 ^th^ percentile) at low protein intensity (20 ^th^ percentile) and the probability of high protein intensity (80 ^th^ percentile) at low velocity magnitude (20 ^th^ percentile) across tissue images. Prior to these calculations, we tested the following cutoffs: 10, 20, 30, and 40%. We determined that the 20% threshold best captured the variability in intensity and velocity, given the spread in intensity and velocity values. This probability approach has allowed us to average probabilities across 10 (Zyxin dataset) or 15 embryos (LPP dataset) across 3 clutches of embryos per group.

## Reagents

**Plasmid DNA**

pCS2+/ *Xenopus laevis* Zyxin-mNeonGreen; This study

pCS2+/ *Xenopus laevis* LPP-mNeonGreen; This study

pCS2+/mRFP-ZO-1; Higashi et al. 2016

pCS2+/TagBFP-ZO-1; Stephenson et al. 2019

pCSf107mT/Lifeact-miRFP703; Yamamoto et al. 2021

pCS2+/PLEKHA7-mCherry; Higashi et al. 2019

***Xenopus laevis* frogs**

*Xenopus laevis* (female), oocyte positive, pigmented; Xenopus 1 or National *Xenopus* Resource (NXR)

*Xenopus laevis* (male), pigmented; Xenopus 1 or National *Xenopus* Resource (NXR)
